# Parental Perception of Body Weight Status of Their 8-year-old Children: Findings from the European CHOP Study

**DOI:** 10.1007/s10995-021-03334-w

**Published:** 2022-01-01

**Authors:** Annick Xhonneux, Jean-Paul Langhendries, Françoise Martin, Laurence Seidel, Adelin Albert, Elena Dain, Martina Totzauer, Veit Grote, Veronica Luque, Ricardo Closa-Monasterolo, Alice Re Dionigi, Elvira Verduci, Darius Gruszfeld, Piotr Socha, Berthold Koletzko, for the European Childhood Obesity Trial Study group

**Affiliations:** 1 Groupe Santé CHC, bd Patience et Beaujonc 2 - (B) 4000, Liège, Belgium; 2 University Hospital (CHU) of Liège, Liège, Belgium; 3 Department of Public Health, University of Liège, Liège, Belgium; 4 Children’s Hospital Queen Fabiola, Université Libre de Bruxelles, Brussels, Belgium; 5 Division of Metabolic and Nutritional Medicine, Dr. Von Hauner Children’s Hospital, University of Munich Medical Centre, Ludwig-Maximilians-Universität Munich, Munich, Germany; 6 Paediatrics, Nutrition and Development Research Unit, Universitat Rovira I Virgili, IISPV, Reus, Spain; 7 Department of Paediatrics, San Paolo Hospital, University of Milan, Milan, Italy; 8 Department of Gastroenterology, Hepatology and Eating Disorders, Children’s Memorial Health Institute, Neonatal Intensive Care Unit, Warsaw Poland and The Children’s Memorial Health Institute, Warsaw, Poland

**Keywords:** Parental child weight perception, Scale of sketches, BMI, Healthy children

## Abstract

**Background:**

Maternal perception of child weight status in children with overweight or obesity has received a lot of attention but data on paternal perception of children from presumably healthy cohorts are lacking.

**Objective:**

We aimed to investigate paternal and maternal perception of child weight status at the age of 8 years in a cohort of 591 children from 5 European countries.

**Material and Methods:**

Included were 8-year-old children and their parents participating in the European Childhood Obesity Project (EU CHOP). Weight and height of children and parents were measured and Body Mass Index (BMI, kg/m^2^) was calculated. Both parents were asked to assess their perception of child weight status using Eckstein scales and their concern about child overweight. The agreement between mother and father perceptions was assessed by Cohen kappa coefficient and their relationship was analyzed by linear mixed effects models based on ordinal logistic regression, accounting for country, child gender and BMI, parental BMI, level of education, concern and type of feeding during first year of life.

**Results:**

Data from children and both parents were available for 432 girls and boys. Mean BMI was comparable in boys and girls (16.7 ± 2.31 vs. 16.9 ± 2.87 kg/m^2^, P = 0.55). In total, 172 children (29.3%) were overweight or obese. There was a high degree of agreement between mother and father perceptions of their child’s weight status (Cohen kappa 0.77). Multivariate modelling showed that perception levels significantly increased with child BMI but were globally lower than assessed. They differed between countries, gender and types of feeding during first year of life, were influenced by education level of the father but were not related to parental BMI and concern about childhood overweight.

**Conclusions:**

The study showed no overall differences between mothers and fathers in rating their child’s weight status but both parents had a propensity to underestimate their child’s actual weight, particularly in boys.

The EU CHOP trial registered at clinicaltrials.gov as NCT00338689.

**Supplementary Information:**

The online version contains supplementary material available at 10.1007/s10995-021-03334-w.

## Significance

Feeding practices have been investigated as potential modifiable risk factors in the etiology of childhood obesity. One factor which could be related to feeding practices is the parental perception of their child’s weight status. This perception, mainly focused in mothers previously, has been studied in already overweight children. Overall, it may be advisable to assess parental perception early enough to avoid their child becoming overweight. We therefore felt it useful to assess this parental perception (mother’s but also father’s one) in the EU CHOP sample as these children initially aimed at reflecting a healthy general population originated from five countries.

## Background

Overweight and obesity in children have dramatically increased over the years, becoming a major public health concern worldwide. According to the World Health Organization (WHO), over 41 million children were overweight or obese in 2016 (WHO | Commission on Ending Childhood Obesity, 2019). The short-term and long-term consequences of childhood overweight have been well-documented (Ebbeling et al., 2002). Genetics, environmental, nutritional, cultural and social factors are major determinants of BMI in childhood (Birch & Davison, 2001). Feeding practices in particular have been investigated as potential modifiable risk factors in the etiology of childhood obesity (Khandpur et al., 2014). One factor which could be related to feeding practices is the parental perception of their child’s weight status (Tabak et al., 2017; Yilmaz et al., 2013). This is especially true for overweight/obese children: a meta-analysis showed that more than half of the parents were reported to underestimate their child’s weight status, regarded as “parental misperception” (Lundahl et al., 2014). Uncertainty exists about factors explaining this misperception (Baughcum et al., 2000; Doolen et al., 2009; Lundahl et al., 2014; Towns & D’Auria, 2009) and implications of this misperception with respect to eating behaviors and obesity prevention (Gerards et al., 2014; McKee et al., 2016; Rietmeijer-Mentink et al., 2013; Robinson & Sutin, 2016). Perception of the child’s weight alone is unlikely to alter parental feeding styles if parents are not concerned about weight (Webber et al., 2010). According to several authors, concern about weight is viewed as an important factor related to feeding practices and childhood obesity (Birch & Davison, 2001; Birch et al., 2001; Crawford et al., 2006; Francis et al., 2001). In this context, most studies focus on mothers’ perception and little is known about fathers’ perception (Doolen et al., 2009; Robinson & Sutin, 2016). Some authors have pointed out the importance of evaluating also paternal feeding practices and father parenting styles in relationship to their child weight status and the risk of overweight and obesity (Blissett et al., 2006; Johannsen et al., 2006; Khandpur et al., 2014; Wake et al., 2007). Therefore, we aimed to characterize both paternal and maternal perception of their child’s weight status in a group of five hundred ninety-one 8-year-old children from five European countries.

## Methods

### Study Population

The present study used data collected in children participating in the European Childhood Obesity Project (EU CHOP), a prospective randomized clinical trial with the primary aim to study effects of infant feeding with different protein intakes on later obesity risk (Koletzko et al., 2009; Weber et al., 2014). Participants of the study were apparently healthy, singleton term infants recruited during the first 8 weeks of life (median age 14 days; IQR 3–30 days) between 1 October 2002 and 31 July 2004 from obstetrical hospital departments in urban areas of five countries (Germany, Belgium, Italy, Poland, and Spain). Children were prospectively followed up to school age (Weber et al., 2014).

### Anthropometric Data and Socioeconomic Status

Information on pregnancy, medical history, socioeconomic status, lifestyle and behavior choices was obtained in standardized parent interviews at baseline visit and when children were 6 years old. The education level of both parents was recorded as none/low, middle or high. Anthropometric measurements, including weight and height, were obtained at baseline and at regular follow-up visits until the age of 8 years and Body Mass Index (BMI) values were calculated (Weber et al., 2014). Standard operating procedures for anthropometric measurements based on the WHO Multicentre Growth Reference Study (de Onis et al., 2004) and repeated training and monitoring sessions were established. The same equipment was used in all centers and regularly calibrated. Overweight and underweight were defined according to WHO references: overweight at 8 years was defined as a BMI at least one standard deviation (SD) above the mean for age (z-score ≥ 1), and underweight as BMI was at least 2 SD below WHO the mean (z-score ≤ -2) (Rolland-Cachera et al., 1991; WHO, n.d.). Obesity was defined based on International Obesity Task Force criteria (Cole et al., 2000) if BMI at 8 years was > 21.57 kg/m^2^ in girls and > 21.60 kg/m^2^ in boys, respectively. Parental anthropometric data were measured by the study staff at baseline and at the child age of 8 years, or self-reported by parents not attending the follow-up visit.

### Parental Perception of Child Weight and Concern Regarding Risk of Overweight

**Fig. 1:**
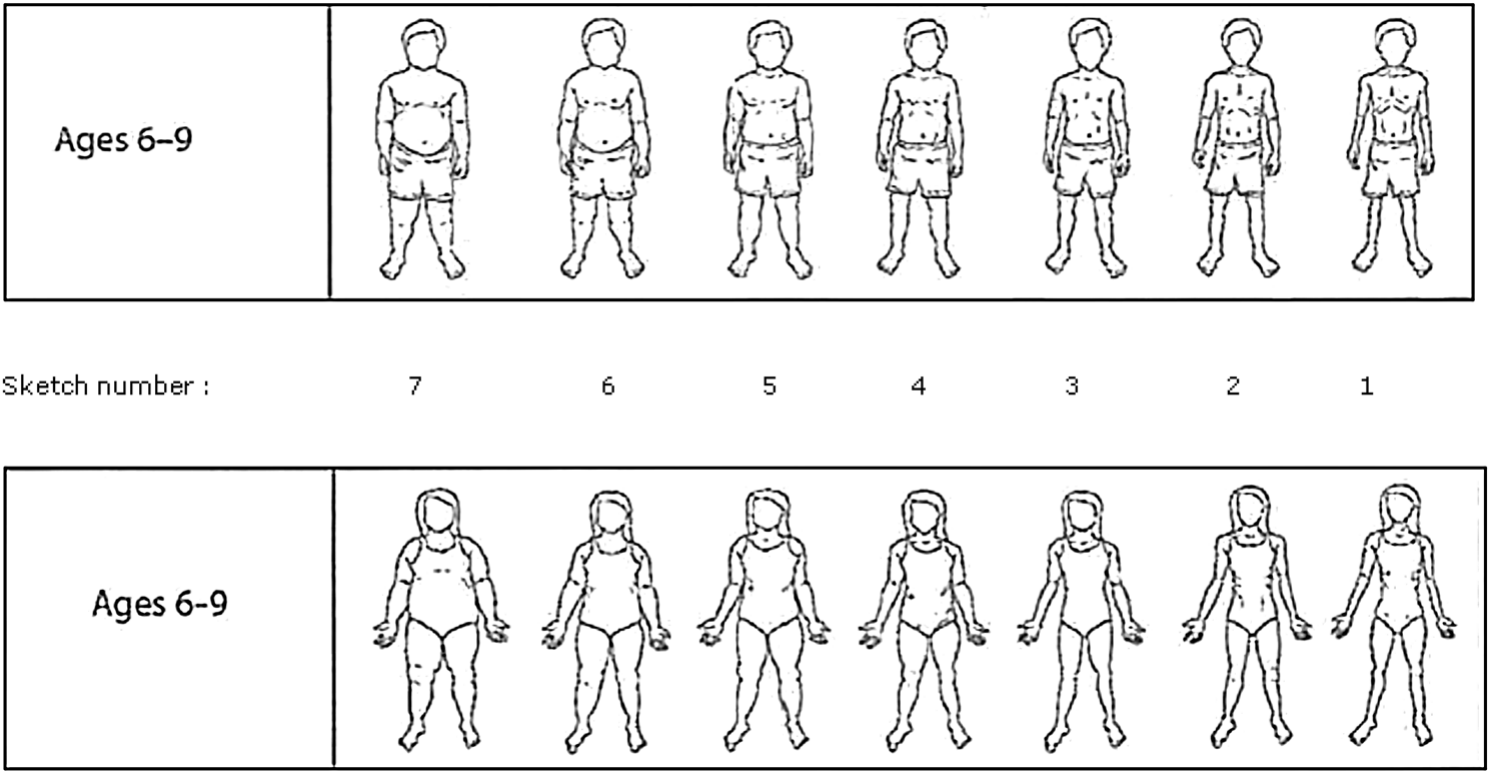
Eckstein scale of sketches (boys and girls, aged 6–9 years) (Production with permission of authors)

We used the sketches scale created by Eckstein & al. (Eckstein et al., 2006) for boys and girls aged 6–9 years to asses parental perception of their child’s weight. The scales show 7 image sketches numbered from 7 (heaviest) to 1 (lightest), with the middle image (sketch number 4) representing a child at 50^th^ percentile BMI (Fig. 1).

Fathers and mothers were asked independently to indicate the picture matching their child’s actual weight status: *“In your opinion, to which sketch does your child most resemble at the moment?”.* They were also asked: *“How concerned are you about your child becoming overweight (not at all, a little, moderate, much, and very much)?”* Parents who answered “much” or “very much” were considered as being highly concerned.

### Statistical Analysis

Quantitative data were summarized as mean ± SD or as median and interquartile range (IQR) in case of skewed distributions while for categorical data frequency tables were used. Linear regression was used to measure the association between a quantitative variable and a set of covariates. The agreement between father and mother perception on the Eckstein scale was based on data available for both parents (N = 432) and assessed by the weighted Cohen kappa coefficient. Similarly, the relationship between parental perception (defined as an ordinal variable) and covariates was evaluated with a generalized linear mixed model based on ordinal logistic regression (GENMOD procedure). Covariates included child’s country, gender, BMI and feeding type during first year of life (breastfed, low or high protein formula), as well as parental features like mother/father BMI, education level and concern about child becoming overweight. Results were considered significant at the 5% significance level (P < 0.05). All statistical analyses were carried out by SAS version 9.4 (SAS Institute, Cary, NC, USA).

## Results

### Children and Parents

The characteristics of the 8-year-old children and their parents where both maternal and paternal perception assessments were available (N = 432) are described in Table 1.

**Table 1:** Characteristics of 8-year-old study children and of their parents (N = 432)

Variable	No. of subjects	Category	Number (%) *
Gender	432	Girl	235 (54.4)
		Boy	197 (45.6)
Country	432	Germany	67 (15.5)
		Belgium	60 (13.9)
		Italy	97 (22.5)
		Poland	68 (15.7)
		Spain	140 (32.4)
Feeding type	432	Low protein	142 (32.9)
		High protein	137 (31.7)
		Breastfed	153 (35.4)
BMI (kg/m^2^)	429		16.8 ± 2.58
Mother education	430	None/low	61 (14.2)
		Middle	221 (51.4)
		High	148 (34.4)
Father education	430	None/low	82 (19.1)
		Middle	220 (51.2)
		High	128 (29.8)
Mother concern	431	Not at all	144 (33.4)
		A little	111 (25.8)
		Moderate	70 (16.2)
		Much/Very much	106 (24.6)
Father concern	429	Not at all	128 (29.8)
		A little	121 (28.2)
		Moderate	85 (19.8)
		Much/Very much	95 (22.1)
Mother BMI (kg/m^2^)	325		25.1 ± 5.07
Father BMI (kg/m^2^)	220		27.1 ± 3.90

*For BMI, summary statistics are mean ± SD

The BMI was comparable in girls and boys (16.7 ± 2.31 vs. 16.9 ± 2.87 kg/m^2^, P = 0.55). According to BMI thresholds, 124 (28.9%) children were overweight (26.7% of girls and 31.4% of boys), 23 (5.4%) obese and 5 (1.2%) were underweight. Most parents (> 90%, data not shown) were born in the same country as their children. There was a highly significant (P < 0.0001) degree of agreement between the education level of fathers and mothers (Cohen κ = 0.50; 95%CI: 0.43–0.57) and similarly between their level of concern (Cohen κ = 0.50; 95%CI: 0.44–0.55). By contrast, the BMI of fathers was higher than the BMI of mothers (P < 0.0001). Among fathers, 65.5% were overweight and 19.5% obese. For mothers, overweight was observed in 43.1% of the cases and obesity in 14.2%.

The relationship between child’s BMI and each covariate separately showed that BMI varied with country (e.g., it was greater in Italy, Poland and Spain compared to Germany) and with feeding type (it was higher for high protein than for low protein or breast feeding during the first year of life). Child BMI was also positively associated with mother/father BMI and level of concern but negatively related to parental education level (see Supplementary Table S1).

### Agreement Between Parental Perceptions

The cross-classification of mother and father perceptions of their child’s weight using the Eckstein scale is displayed in Table 2. Globally, 303 of 432 parental assessments (70.1%) were concordant, with a high overall agreement between both parents (Cohen κ = 0.73; 95%CI: 0.68–0.77) to a similar extent for boys (κ = 0.74) and girls (κ = 0.71).

**Table 2:** Cross-classification of mother and father perception of child weight status using the Eckstein scale (1 = lightest, ≥ 6 heaviest) based on 432 couples

	Father’s choice						
Mother’s choice	1	2	3	4	5	≥ 6	Total (%)
1	17	14	2	0	0	0	33 (7.6)
2	8	94	34	0	0	0	136 (31.5)
3	0	28	138	6	1	0	173 (40.1)
4	0	0	16	30	8	0	54 (12.5)
5	0	0	0	5	13	3	21 (4.9)
≥ 6	0	0	1	0	3	11	15 (3.5)
Total (%)	25 (5.8)	136 (31.5)	191 (44.2)	41 (9.5)	25 (5.8)	14 (3.2)	N = 432

**Fig. 2:**
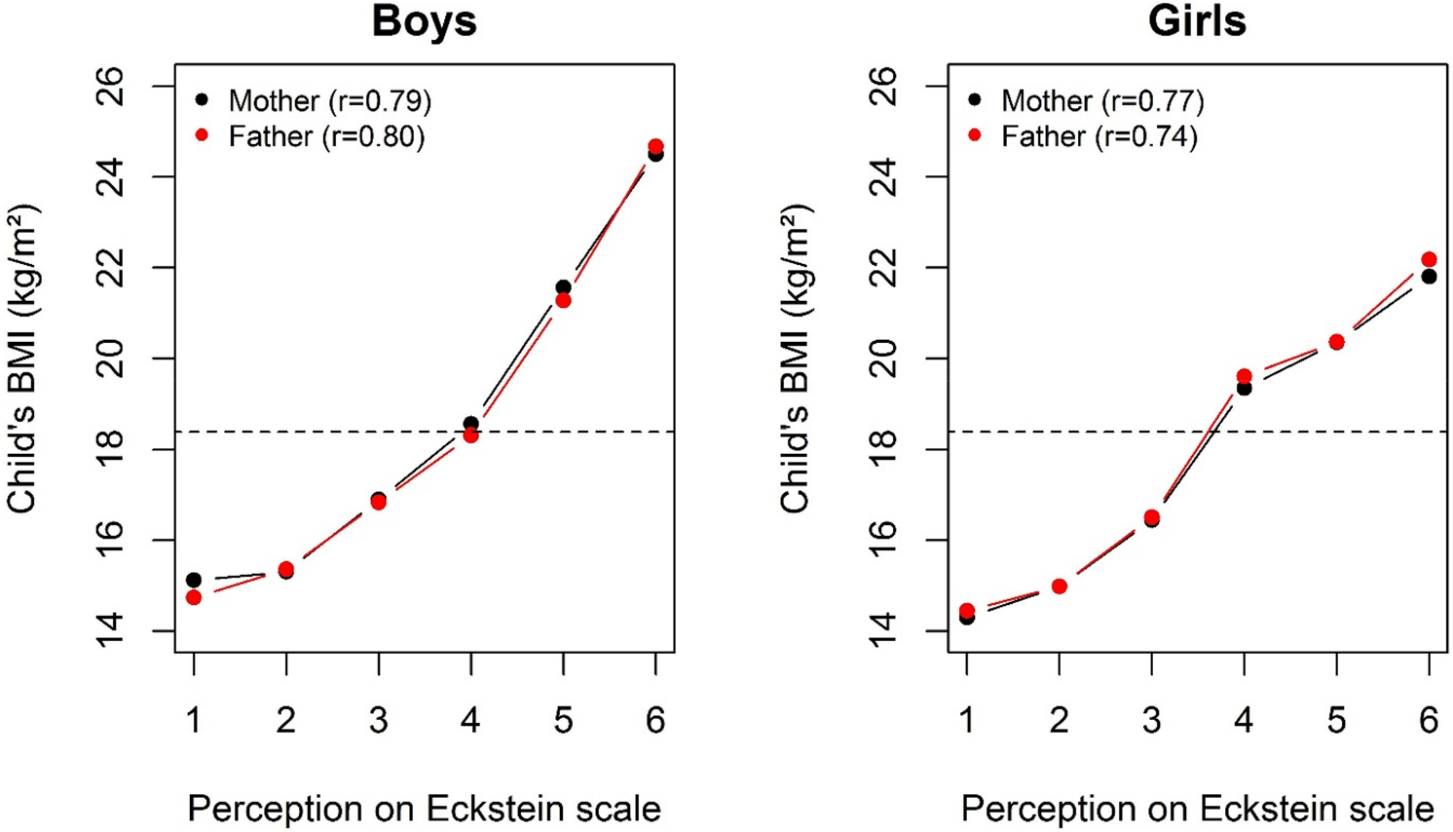
Child BMI mean values according to mother and father perception of body weight status in boys and girls (the dotted line represents the IOTF threshold for overweight)

When looking at the relationship between mean child BMI and parental perception ratings, curves of mothers and fathers were perfectly superimposed for boys as well as for girls (Fig. 2). Since on both graphs the perception category “4” represents the median BMI and the dotted horizontal line the “IOFT threshold” of overweight, it is concluded that both parents had a propensity to underestimate their child’s weight status regardless of child’s gender but more strongly in boys than in girls.

### Factors Associated with Parental Perceptions

To assess the association of parental perception assessment of child’s weight status with child-related and parent-related covariates, a generalized linear mixed effects model was fitted to the data. Results expressed as regression coefficients with standard errors and P-values are displayed in Table 3.

**Table 3:** Association of parental perception scoring of child’s weight status on the Eckstein scale (1–7) and child-related and parent-related factors in 432 children and their parents

Covariate	Category	Coefficient ± SE*	P-value
Child’s gender	Girl	0.0	NA
	Boy	− 0.72 ± 0.18	< 0.0001
Child’s country	Germany	0.0	NA
	Belgium	− 0.029 ± 0.32	0.93
	Italy	0.46 ± 0.27	0.090
	Poland	0.71 ± 0.30	0.016
	Spain	0.20 ± 0.28	0.48
Child’s BMI (kg/m^2^)		1.02 ± 0.071	< 0.0001
Child’s parent	Father	0.0	NA
	Mother	0.58 ± 0.38	0.13
Mother education level	Low	0.0	NA
	Middle	− 0.20 ± 0.34	0.54
	High	0.14 ± 0.37	0.70
Father education level	Low	0.0	NA
	Middle	0.81 ± 0.27	0.0030
	High	0.81 ± 0.30	0.0061
Mother concern level	Not at all	0.0	NA
	A little	− 0.005 ± 0.25	0.98
	Moderate	0.57 ± 0.36	0.11
	Much/very much	0.46 ± 0.31	0.13
Father concern level	Not at all	0.0	NA
	A little	0.063 ± 0.26	0.81
	Moderate	0.34 ± 0.31	0.27
	Much/very much	0.016 ± 0.31	0.96
Child’s feeding type	Low protein	0.0	NA
	High protein	0.095 ± 0.23	0.68
	Breastfed	0.62 ± 0.23	0.0070

*Positive (negative) coefficients indicate a higher (lower) probability to perceive the child’s weight status at heavier (lighter) sketches

Results confirm that, when accounting for child’s BMI, parental perception ratings were lower for boys, different between countries with significantly higher grades assigned by Polish than German parents, and comparable among parents (P = 0.13). Further, a highly significant association was noted with education level of fathers but not of mothers. Fathers with middle/high education levels attributed higher perception ratings compared to fathers with a low education level. As for the level of concern about child overweight risk, no association was evidenced for either parent. Finally, parents of previously breastfed children rated their children with higher ratings than those of previously formula fed children. Most findings were confirmed by including parental BMI in the model despite a substantial loss of observations due to missing data (Supplementary Table S2).

## Discussion

### Main Findings

This study showed a high degree of agreement between maternal and paternal perception of their child’s body weight when data were collected concomitantly. Perception remains a theoretical topic of interest in relation to the prevention of child overweight. To our knowledge, most previous studies on perception have collected information on the primary caregivers of the child, who could be the mother or the father. Clearly, taking into account the opinion and perception of fathers can be justified by the increasing involvement of fathers in the household (e.g. feeding their children) as more and more mothers have a professional occupation outside home (Khandpur et al., 2014)(Hudson et al., 2012). In the present study, data were collected simultaneously for both parents and compared. This comparison showed that the perception of the child’s weight by fathers strongly agreed with that of the mothers (Cohen κ = 0.73).

We also found a propensity of both parents to underestimate their child’s weight, particularly for boys. This finding is in line with those of Lundhal who reported that mothers of healthy children tended to underestimate their child’s weight (Lundahl et al., 2014). One explanation could be that parents, living in an environment where many children and adults are overweight or obese, may develop inaccurate perceptions and tend to consider their children thinner than the average population (Maximova et al., 2008).

Several factors were found to be associated with parental rating, including child gender, country of living, and parental education level. In the present study, the percentages of normal and overweight girls were similar to those found for boys. Boys, however, had a higher probability of being classified in lower classes of sketches than girls. This potential influence of child gender was already reported in other studies (Lundahl et al., 2014; Towns & D’Auria, 2009). One study (Maynard et al., 2003) showed that the risk for girls of being classified in a higher category of weight was 3 times higher than for boys. Mamun et al. (Mamun et al., 2008) reported that the body perception of daughters with a normal weight or overweight was better evaluated than the body perception of boys. More recently, another study confirmed that child’s gender could influence parental perception (Hudson et al., 2012).

Demographic criteria such as parental education are often explored in studies related to perception. Maternal education was one of most consistently influencing factors reported (Baughcum et al., 2000), although it was not always significant (Maximova et al., 2008; Tabak et al., 2017). Most of the populations studied were overweight children, in contrast to our study. We found differences between mothers and fathers regarding the influence of education level. This deserves further evaluation, considering that parental education level is a strong predictor of child overweight risk (Cullinan & Cawley, 2017; Muthuri et al., 2016).

Parental perception was different between participating countries, with Polish parents giving ratings that were higher than those of parents in other countries. While the influence of cultural background on parental perception of child weight has been studied in the USA (Contento et al., 2003; Sherry et al., 2004), data on this question are still lacking for European countries (Gualdi-Russo et al., 2012; Manios et al., 2009; Remmers et al., 2014). We suggest this issue be investigated in more detail.

Parental concern about child weight was significantly positively associated with the child’s BMI. However, only 20% of the parents indicated a high level of concern over their child becoming overweight, while the percentage of overweight children was around 30%. Maynard et al. (Maynard et al., 2003) reported that parental level of health-related concern for their child was more closely associated with perception of their child’s activity level than their weight status. Parents appear not to worry much about their children’s long term health and tend to consider their age as too young to develop overweight-related problems (Towns & D’Auria, 2009). Of interest and contrary to our expectations, the level of concern did not statistically influence parental perception. One explanation could be that concern and perception should be studied as two different mechanisms relating child weight and parental feeding practices; mothers appear to adopt specific restrictive feeding strategies in response to their concerns about their child weight which is not mediated by perception. In all cases, both variables were associated with readiness of parents to make changes to help their overweight child losing weight (Rhee et al., 2005). As, in general, parental perception of the weight status of an already overweight child will not prevent weight gain, it may be advisable to assess parental perception early enough to avoid their child becoming overweight (Parkinson et al., 2017). However, many parents tend to classify their child as overweight only in case of more advanced overweight, when it is more difficult to implement effective weight-related actions. Prevention of obesity is facilitated by appropriate actions in the early phase of excessive weight gain, when parents with support of healthcare providers can address modifiable risk factors such as dietary habits and physical activity.

## Strengths and Limitations

The fact that the study was based on the Eckstein scale can be seen as a strong asset. The Eckstein scale offers advantages over several other tools suggested to evaluate parental perception of child weight status, such as growth references not recognized by mothers as relevant (Jain et al., 2001), written questions (Baughcum et al., 2000; Gerards et al., 2014; Mamun et al., 2008; Maynard et al., 2003; Remmers et al., 2014), or different other visual scales for evaluating parental perception of body figure (Contento et al., 2003; Huang et al., 2007; Maximova et al., 2008; Saxton et al., 2009; Truby & Paxton, 2002; Yilmaz et al., 2013). The Eckstein scale is simple and easy-to-use for parents, the pictograms are more appealing than items in a questionnaire, they are available for both genders at different ages, neutral to race and ethnicity and not inclined towards underestimation by parents (Lundahl et al., 2014). Another strong point of this study is the prospective and standardized data collection and the inclusion of families with healthy children of the same age across five different countries, using the same methodological approach. Data analyses were adjusted for potentially influencing factors. Among limitations, there is the risk for selection bias since participation in the CHOP study was voluntary and may have an underrepresentation of less privileged groups of society. Since the CHOP study’s primary objective was to explore the effect of infant feeding on growth, parents who agreed to participate might have been more concerned about their child risk of obesity than the general population. In fact, the proportion of overweight mothers in the CHOP study was lower than the average rates reported in the 5 participating countries (Health Organization & Office for Europe, R., 2013), but mothers were on average younger than expected in the population, which may contribute to parts of the difference. In contrast, the proportion of overweight fathers in the study was similar to that reported in the respective European countries. Another limitation concerned missing data since rating of body weight perception by both parents was not available for all children. The same problem could be pointed out for the parental BMI which unfortunately was lacking for several couples.

## Conclusions

For the first time, father and mother perception of their own child’s body weight status was compared in a large cohort of healthy children across several countries. No difference was highlighted between fathers and mothers but both parents had a tendency to underestimate their child’s actual weight status, in particular for boys. The study also pointed out the need to inform parents about their children weight status and the potential risks associated. Specifically, further research should explore how parental perception and concern are related to feeding practices and adiposity risk.

## Supplementary Information

Below is the link to the electronic supplementary material.

Supplementary file1 (DOCX 20 KB)

## Data Availability

The CHOP cohorts’ data are available only to the collaborating scientists from the respective CHOP participating centers. The data may be available upon request for some of the participating centers but not for all due to relevant data protection laws.

## Abbreviations

BMI: Body Mass Index
EU CHOP: EU Childhood Obesity Project
HP: Group of children fed cow’s-milk formula with higher protein content
LP: Group of children fed cow’s-milk formula with lower protein content
BF: Breastfed infants (control group)
SD: Standard deviation
SE: Standard error
WHO: World Health Organization
