# Supplementary material for: Parental Perception of Body Weight Status of Their 8-year-old Children: Findings from the European CHOP Study
Source: Matern Child Health J. 2022 Jan 1;26(6):1274–82. doi: 10.1007/s10995-021-03334-w (PMC9132811; doi:10.1007/s10995-021-03334-w)
Supplement: Supplementary file 1 — Supplementary file1 (DOCX 20 KB) [file 10995_2021_3334_MOESM1_ESM.docx]

Table S1. Relationship between child’s BMI and each child/parent-related characteristic (n= 432)

| Variable | Category | Coefficient ± SE | P-value |
| --- | --- | --- | --- |
| Gender | Boy | 0.15 (0.25) | 0.56 |
| Country | Belgium | -0.013 ± 0.45 | <0.0001 |
| (vs. Germany) | Italy | 1.50 ± 0.40 |  |
|  | Poland | 1.17 ± 0.44 |  |
|  | Spain | 1.24 ± 0.38 |  |
|  |  |  |  |
| Feeding type | High protein | 0.60 ± 0.31 | 0.0058 |
| (vs. Low protein) | Breastfed | -0.37 ± 0.30 |  |
|  |  |  |  |
| Mother education | Middle | -0.76 ± 0.37 | 0.016 |
| (vs. None/low) | High | -1.12 ± 0.39 |  |
|  |  |  |  |
| Father education | Middle | -0.27 ± 0.33 | 0.0004 |
| (vs. None/low) | High | -1.24 ± 0.36 |  |
|  |  |  |  |
| Mother concern | A little | 1.08 ± 0.29 | <0.0001 |
| (vs. Not at all) | Moderate | 2.60 ± 0.33 |  |
|  | Much/very much | 3.01 ± 0.29 |  |
|  |  |  |  |
| Father concern | A little | 0.79 ± 0.30 | <0.0001 |
| (vs. Not at all) | Moderate | 2.51 ± 0.33 |  |
|  | Much/very much | 2.70 ± 0.32 |  |
|  |  |  |  |
| Mother BMI* |  | 0.16 ± 0.03 | <0.0001 |
| Father BMI* |  | 0.26 ± 0.04 | <0.0001 |

*Mother BMI and father BMI available for only 325 and 220 subjects, respectively. Positive (negative) coefficients indicate an increasing (decreasing) relationship with child’s BMI

Table S2. Association of parental child weight status rating on the Eckstein scale (1-7) and child-related and parent-related factors including parental BMI (N=177)

| Covariate | Category | Coefficient ± SE* | P-value |
| --- | --- | --- | --- |
| Gender | Girl | 0.0 | NA |
|  | Boy | -0.71 ± 0.22 | 0.0010 |
|  |  |  |  |
| Country | Germany | 0.0 | NA |
|  | Belgium | -0.071 ± 0.36 | 0.84 |
|  | Italy | 0.56 ± 0.33 | 0.083 |
|  | Poland | 0.85 ± 0.38 | 0.026 |
|  | Spain | 0.25 ± 0.35 | 0.48 |
|  |  |  |  |
| BMI (kg/m²) |  | 1.11 ± 0.095 | <0.0001 |
|  |  |  |  |
| Parent | Father | 0.0 | NA |
|  | Mother | 2.43 ± 1.36 | 0.074 |
|  |  |  |  |
| Mother education level | Low | 0.0 | NA |
|  | Middle | -0.49 ± 0.38 | 0.20 |
|  | High | -0.064 ± 0.42 | 0.88 |
|  |  |  |  |
| Father education level | Low | 0.0 | NA |
|  | Middle | 0.78 ± 0.41 | 0.058 |
|  | High | 0.89 ± 0.44 | 0.044 |
|  |  |  |  |
| Mother concern level | Not at all | 0.0 | NA |
|  | A little | -0.040 ± 0.31 | 0.90 |
|  | Moderate | 0.50 ± 0.42 | 0.24 |
|  | Much/very much | 0.081 ± 0.40 | 0.84 |
|  |  |  |  |
| Father concern level | Not at all | 0.0 | NA |
|  | A little | 0.092 ± 0.38 | 0.81 |
|  | Moderate | 0.22 ± 0.41 | 0.59 |
|  | Much/very much | -0.022 ± 0.42 | 0.96 |
|  |  |  |  |
| Mother BMI (kg/m²) |  | -0.042 ± 0.024 | 0.078 |
| Father BMI (kg/m²) |  | 0.016 ± 0.038 | 0.67 |
|  |  |  |  |
| Feeding type | Low protein | 0.0 | NA |
|  | High protein | 0.055 ± 0.27 | 0.84 |
|  | Breastfed | 0.56 ± 0.28 | 0.047 |

*Positive (negative) coefficients indicate a higher (lower) probability to perceive the child’s weight status at heavier (lighter) sketches
